# Transporting Ocean Viromes: Invasion of the Aquatic Biosphere

**DOI:** 10.1371/journal.pone.0152671

**Published:** 2016-04-07

**Authors:** Yiseul Kim, Tiong Gim Aw, Joan B. Rose

**Affiliations:** 1 Department of Microbiology and Molecular Genetics, Michigan State University, East Lansing, Michigan, United States of America; 2 Department of Fisheries and Wildlife, Michigan State University, East Lansing, Michigan, United States of America; University of Connecticut, UNITED STATES

## Abstract

Studies of marine viromes (viral metagenomes) have revealed that DNA viruses are highly diverse and exhibit biogeographic patterns. However, little is known about the diversity of RNA viruses, which are mostly composed of eukaryotic viruses, and their biogeographic patterns in the oceans. A growth in global commerce and maritime traffic may accelerate spread of diverse and non-cosmopolitan DNA viruses and potentially RNA viruses from one part of the world to another. Here, we demonstrated through metagenomic analyses that failure to comply with mid-ocean ballast water exchange regulation could result in movement of viromes including both DNA viruses and RNA viruses (including potential viral pathogens) unique to geographic and environmental niches. Furthermore, our results showed that virus richness (known and unknown viruses) in ballast water is associated with distance between ballast water exchange location and its nearest shoreline as well as length of water storage time in ballast tanks (voyage duration). However, richness of only known viruses is governed by local environmental conditions and different viral groups have different responses to environmental variation. Overall, these results identified ballast water as a factor contributing to ocean virome transport and potentially increased exposure of the aquatic bioshpere to viral invasion.

## Introduction

Viruses are the most undiscovered and mysterious part of the biosphere. Their role as pathogenic entities is well recognized and the array of viral infections throughout the tree of life, including archaea, bacteria, and eukaryotes, is immense. However, we have only scratched the surface to reveal the global genetic diversity of viruses. This has limited our understanding of the ecological role of phages and other viral groups in biogeochemical cycling, as well as gene exchange [1]. Our knowledge of the viral predator-prey interactions is poor and viral life histories have not been well described. Viral-host specificity that was once considered a well-known biological principal is now being challenged, as even the concept of plant viral infections of humans and other animals is being proposed [2].

During the past decade, metagenomics with dramatic evolution of sequencing technologies have revolutionized environmental virological studies and enabled the in-depth characterization of viral communities that would not have been possible with traditional methods. Since the first viral metagenome (virome) study by Breitbart *et al*. [3], research has demonstrated the feasibility of metagenomic approaches to examine viral communities in various complex environmental systems, mostly focused on natural aquatic environments, marine [4–10] and freshwater [11–17]. Among these, two global surveys of the ocean virome, which focused mainly on DNA viruses infecting bacteria, have suggested that marine viruses, particularly phages are highly diverse and can exhibit distinctive biogeographic patterns [4,10]. While these studies have revealed a diverse array of DNA phages (e.g., *Microviridae*, *Myoviridae*, *Podoviridae*, and *Siphoviridae*) in marine environments and that local environmental conditions play an important role in structuring their diversity, little is known about the diversity of RNA viruses and eukaryotic viruses in the oceans and their global transport and disease potential.

Oceanic and coastal anthropogenic pollution is growing in part as a function of global commerce and increasing maritime traffic. It is estimated that ocean-going cargo vessels transport as high as 12 billion tons of ballast water each year, transferring the aquatic life from one part of the world to another [18]. Global movement of nonindigenous species within ballast tanks across natural barriers has threatened coastal ecosystem and biodiversity. The metazoan ballast invaders have been well studied and described since about the 1980s [19,20]. However, the mechanisms of microbial invasions are still unclear despite the potential of microorganisms to influence the ecological functioning of biological communities and ecosystems at a global scale [21]. Ruiz *et al*. [22] provided a hypothesis that the likelihood of invasions goes up with increasing inoculation concentration and that genetic diversity of the microbial component in ballast water including viruses must be examined to further understand the global transport of pathogens. More than a decade later, this call to improve our scientific knowledge has remained unanswered despite the advancement of metagenomics using high-throughput sequencing. Here, we integrated environmental virology, metagenomics, and bioinformatics to examine variation in virome composition of ballast water between geographic locations and demonstrated that ballast water moves around ocean viromes (including potential viral pathogens) from one part of the world to another.

## Materials and Methods

### Ethics Statement

Access to the Port of Los Angeles/Long Beach (LA/LB) was gained by California State Lands Commission, and the ballast water sampling was approved by the captains of vessels. Access to the Port of Singapore was gained by Port of Singapore Authority, and the ballast water sampling was approved by an anonymous shipping company and by the captains of vessels. At both locations, the sampling was conducted under the supervision of the captains and chief officers of vessels. Samples collected from the Port of Singapore were transported to Michigan State University (MSU) with the import permit approved by United States Centers for Disease Control and Prevention. Names of vessels were designated as random letters as part of the sample confidentiality agreement.

### Sample collection

A total of 14 samples were collected from the Port of LA/LB, including 11 ballast waters and three surface harbor waters over a one-week period on March 2014 (S1 Table). Samples were transported to a lab in the Cabrillo Marine Aquarium in San Pedro, CA and processed within 12 h of sample collection. Additional 10 samples were collected from the Port of Singapore, including five ballast waters and five surface harbor waters over a two-week period on May 2014. Samples were transported to a lab in National University of Singapore, Singapore and processed within 12 h of sample collection. Type of vessels whose ballast waters were sampled included container ship (8), bulk carrier (3), tanker ship (1), car carrier (1), cruise ship (1), and refrigerated cargo carrier (1). For sample collection, ballast waters were sampled mainly through ballast tank manholes (14 samples). When an access to ballast tank manholes was not available, samples were collected via ballast water pipelines (two samples). Prefix ‘C’ and ‘S’ were used to differentiate samples collected from the Port of LA/LB (e.g., CADO) and the Port of Singapore (e.g., SCB), respectively.

### Variable estimation

Background environmental conditions, including pH, salinity, and temperature of ballast and harbor waters were measured on site using a hand-held meter (model 63, Yellow Springs Instruments, Yellow Springs, OH, USA) and turbidity using a portable meter (model 2020we, LaMotte Company, Chestertown, MD, USA). Ballast water storage duration was calculated based on the difference in days the ballast water was held in the tanks before sample collection. Surface harbor waters were considered to have storage duration of zero-day. Ballast water management practice, replacement of ballast water taken up from a port of origin with water from the open ocean was conducted by 15 out of 16 vessels prior to ballast water discharge either in the Port of LA/LB or the Port of Singapore. Thus, locations of ballast water exchange of 15 vessels and the last port of one vessel carrying unexchanged ballast water were used as geographic origins of ballast water. Coordinates of ballast water exchange location were retreived from ballast water reporting form under the permission of captains of vessels. Distance in nautical miles between where ballast water exchange took place and nearest shoreline was calculated using a data set (http://oceancolor.gsfc.nasa.gov/DOCS/DistFromCoast/) generated by National Aeronautics and Space Administration Ocean Color Group.

### Virome generation

Virome generation was performed following the procedure described in an earlier publication [16]. In brief, viral particles in approximately 60 liters of each sample were concentrated using 30 kDa tangential flow filter (REXEED 25S, Asahi Kasei Medical Co., Ltd., Tokyo, Japan). For samples collected from the Port of LA/LB, the concentrate (300–500 ml) was transported overnight to MSU at 4°C. Viral particles were further concentrated and purified using PEG precipitation, by mixing the concentrate (pH adjusted to 7.2) with 10% PEG 8000 (w/v) and 0.3 M NaCl [23]. After incubation of the mixture at 4°C for 18 h followed by centrifugation at 11,300g for 30 min, the resulting pellet was dissolved in 20 ml of phosphate buffer saline (PBS, pH 7.2). For samples collected from the Port of Singapore, the PEG concentrate (20 ml) was transported to MSU at 4°C. Additional viral purification was performed by adding chloroform (1 volume) to the PEG concentrate and the mixture was centrifuged at 3,000g for 30 min. The aqueous layer was passed through 0.22 μm filters and stored at -80°C. Prior to viral nucleic acid extraction, each 0.22 μm filtrate was treated with DNase I (final concentration of 100 U for 2 h at room temperature) and inactivated with EDTA (final concentration of 8 mM (pH 8.0) for 15 min at 75°C).

Viral nucleic acids were then extracted in three technical replicates for each sample to minimize variation in virome preparation (QIAamp MinElute Virus Spin Kit, Qiagen, Valencia, CA, USA). To confirm the absence of microbial contamination, an aliquot from all samples was screened by 16S rDNA PCR. Following this, samples were again passed through a 0.22 μm filter and treated with DNase I if microbial contamination was detected. To generate sufficient material for Illumina library construction, a random reverse transcription/amplification protocol was used to amplify both viral DNA and RNA [24]. Three separate reactions were performed for each viral nucleic acid extract to minimize potential bias in amplification. The amplified products from each sample were subsequently pooled and purified using PCR Clean-Up System (Promega, Madison, WI, USA).

### Illumina sequencing

The sequencing libraries of 72 samples were prepared using the Illumina TruSeq Nano DNA Library Preparation Kit with few modifications at the Research Technology Support Facility at MSU. The resulting libraries (200-base pair (bp) insert + 120-bp adapters) were loaded on Illumina HiSeq 2500 Rapid Run flow cells and sequencing was performed in a 2 × 100 bp paired-end (PE) format.

### Bioinformatic analysis of DNA and RNA viromes

We performed quality control by removing (i) reads homologous to a 17-bp sequence (GTTTCCCAGTCACGATC) used as a primer for random transcription/amplification (allowing up to 3 mismatches per read) and (ii) low quality reads (defined as reads < 30 bp in length, with quality score of 50% of the bases < Q30, and/or with degenerate bases (‘N’s)). Finally, we generated 13.3–174.7 million high quality reads for the 72 samples, with an average of 47 million reads per sample.

Sequence reads were assembled into contiguous reads (contigs) using IDBA-UD [25]. Alignment of reads to contigs was also performed with Bowtie 2 [26]. A total of 7.0 million contigs were produced for the 72 samples, and average 79.7 ± 8.1% of reads were mapped at a unique position of the contigs. We carried out taxonomic assignment of contigs by performing BLASTX searches (E <10^−5^) against sequences in the National Center for Biotechnology Information (NCBI) viral database (downloaded in September 2014), and then summarizing the results with MEGAN (Min Score = 50.0, Max Expected = 1.0E^-5^, Top Percent = 10.0, Min Support Percent = 0.0, Min Support = 1, and LCA Percent = 100.0) [27]. Of assigned contigs (2.17 million), we removed contigs that lacked any taxonomic information (e.g., unclassified phages) from the data sets. The abundance of a viral taxonomic group was determined by *Ri = Σ (Ni/Li)*, where *Ri* is the relative abundance of viral family i, *Ni* is the number of reads aligned to a contig in viral family i, and *Li* is the length (kbp) of a contig in viral family i. To compare a particular group of viruses in a virome to the rest of the viromes and to normalize different sequencing scale between viromes, the percentage of the relative abundance of a phylogenetic group within a virome was used rather than its raw value. Information on the relative abundance of viral taxonomic group was compiled in a matrix where different viromes were represented as rows and taxonomic groups in columns. Similarity Percentages (SIMPER) analysis was performed to identify discriminating taxonomic groups by comparing relative abundances of viral families between geographic origins using PAST statistical package [28]. Spearman's correlation coefficient was computed to examine relationships between discriminating viral families and geographical locations using R Statistics Environment [29].

A subset of contigs most similar to viruses infecting human, fish, and shrimp were extracted from the data sets. These contigs were again BLASTX-searched (E <10^−3^) against the inclusive NCBI non-redundant (nr) database (downloaded in April 2014) and any contigs more similar to non-viral proteins were excluded. Genome coverage plots were computed for the selected viral pathogens to examine predicted genes similar to each gene on the reference genomes from the NCBI viral database using Metavir 2 [30].

We used two approaches to estimate the total number of distinct viral species (viral richness) present in each of our viromes. First, we defined virus richness as a total number of identified viral families in the data sets. As relying on the assigned taxonomic groups to determine viral richness limits the observation of unassigned viral groups, tools specifically designed to calculate viral richness (known and unknown viruses) were used as our second approach. Briefly, 2,500,000 quality trimmed reads were randomly sampled from each virome data sets. Contig spectra was calculated with Circonspect [4] using the Minimo assembler employing default parameters (98% sequence identity overlapping by at least 35 bp) on all reads. Then, CatchAll [31] was employed with its default parameters and produced viral richness estimates under the best parametric model according to statistical and heuristic criteria. Spearman's correlation coefficient was computed to examine relationships between virus richness and variables using R Statistics Environment [29].

To take all sequences into account in virome comparison rather than a small known fraction with the use of publically available sequence databases, sequence similarity was computed using TBLASTX comparison as implemented in Metavir 2 [30]. Briefly, a subset of 2,500,000 quality trimmed reads from each virome was uploaded to Metavir 2. Assembled contigs were not used for virome-to-virome comparison, as assembly step introduces bias in the relative abundance of each sequence. The average of best TBLASTX hit scores between virome A reads and virome B reads was computed to represent the sequence similarity between viromes. The resulting similarity matrix (through 0 for no similarity to 100 for a perfect match) for all virome pairs was converted to a dissimilarity matrix by subtracting from 100. A heatmap was generated by a hierarchical cluster analysis using the complete linkage algorithm in R Statistics Environment [29]. To test for statistically significant differences between groupings of the samples made according to geographic origins, Analysis of similarity (ANOSIM) (9999 permutations) was carried out on the previously generated dissimilarity matrix using PAST statistical package [28].

### Data deposition

Virome data sets for all samples have been deposited in the NCBI Short Read Archive under accession number SRP061842.

## Results and Discussion

### Influence of global shipping on transport of the ocean virome

**Fig 1:**
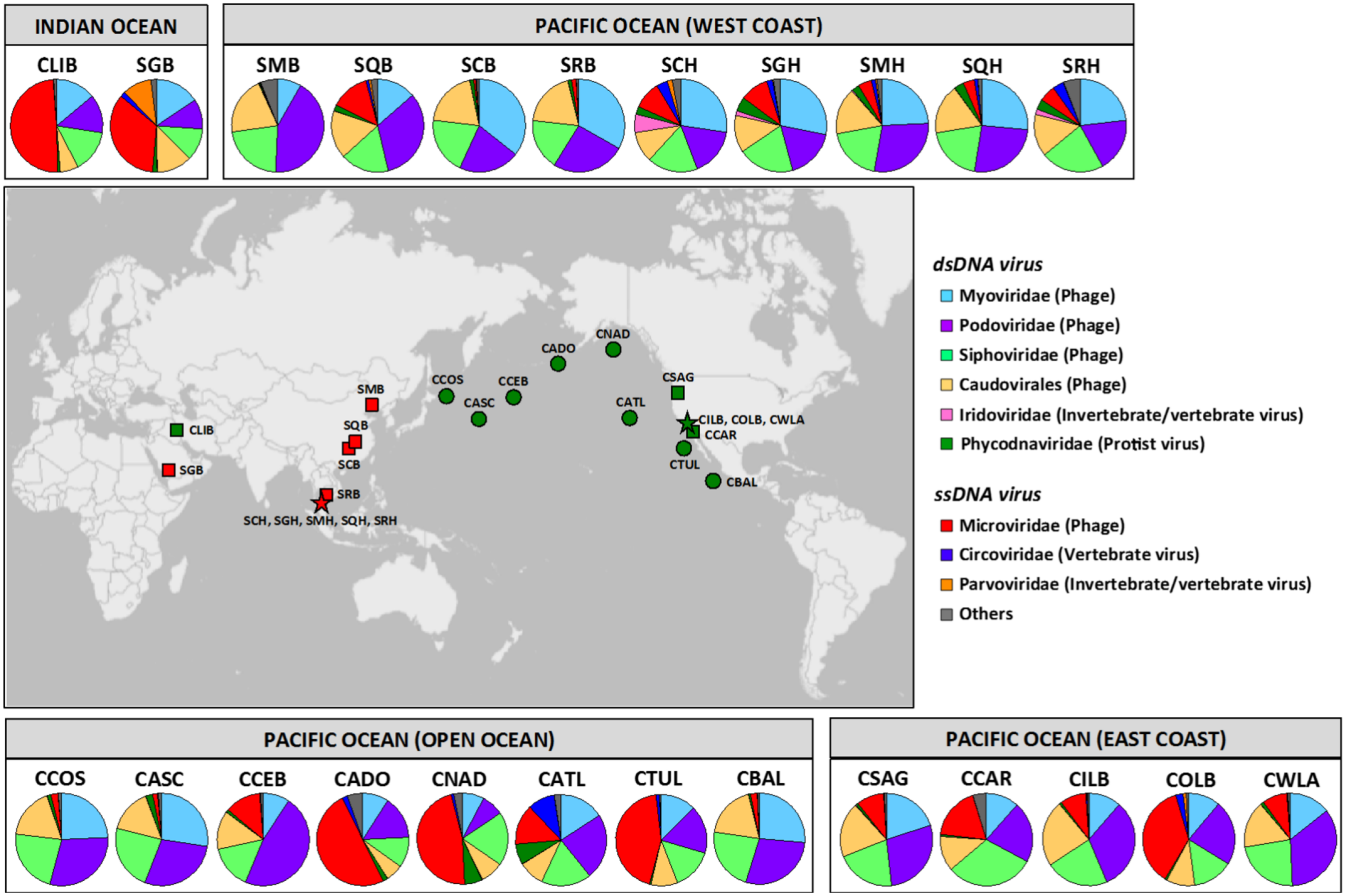
Relative distribution of viromes from ballast and harbor waters. Pie charts represent a mean relative abundance of viral families (three replicates from 24 samples). ‘Others’ are viral families whose maximum relative abundances across viromes are less than 3% (including RNA viruses). Vessels with ballast waters arriving in the Port of LA/LB are shown as a green star and the Port of Singapore as a red star. Circles and squares in the map indicate ballast waters exchanged beyond and within 200 nautical miles from nearest shoreline, respectively. ds, double-stranded; ss, single-stranded.

We explored viral communities in 24 ocean-captured ballast and harbor waters at two distinct geographic locations, the Port of LA/LB and the Port of Singapore, among the world's busiest container ports (Fig 1 and S1 Table). We minimized a potential bias in virome preparation by generating three technical replicates for each sample, which contained concentrated and purified viral particles. The resulting 72 ballast and harbor water virome data sets comprised 3.8 billion 100-bp PE Illumina reads with an average of 52.2 ± 30.9 (mean ± s.d.) million reads (S2 Table). Our virome data sets captured genomes of both DNA and RNA viruses present in ballast and harbor waters.

Here, we first narrowed our focus on taxonomically describable viruses in ballast and harbor waters. To increase the probability of obtaining a significant similarity with reference sequences in the NCBI viral database, 3.4 billion high quality reads of the 72 samples were assembled, generating a total of 7.0 million contigs with an average of 97,357 ± 57,922 contigs with a mean length of 696.7 bp. As reported in other virome studies of marine environment [4,7–9], but not limited to, our BLASTX searches (E < 10^−5^) against the reference sequences revealed the enormous genetic diversity of viruses in the oceans, which cannot be uncovered using publicly available sequence database. Among the contigs homologous to known viruses (30.6 ± 0.03%), the majority was associated with double-stranded (ds) DNA phages (*Myoviridae*, 18.8 ± 8.4%; *Podoviridae*, 24.6 ± 9.5%; *Siphoviridae*, 19.1 ± 4.4%; and unclassified *Caudovirales*, 14.4 ± 4.7%) followed by single-stranded (ss) DNA phage, *Microviridae* (16.3% ± 17.0%). Along with phages, viruses infecting a broad range of hosts, including archaea, fungi, invertebrate, plant, protist, and vertebrate were present at different abundances in our viromes (S3 Table).

Although a higher relative abundance of DNA viruses was found in our virome data sets, 40 viral families were detected as homologous to RNA viruses (9 dsRNA viruses, 31 ssRNA viruses) among 83 viral families (S3 Table). The majority of these RNA viruses (38 families) were found to infect eukaryotic domain, mostly plants, vertebrates, and invertebrates. The other two RNA viral families, *Cystoviridae* and *Leviviridae*, infect prokaryotic domains.

**Fig 2:**
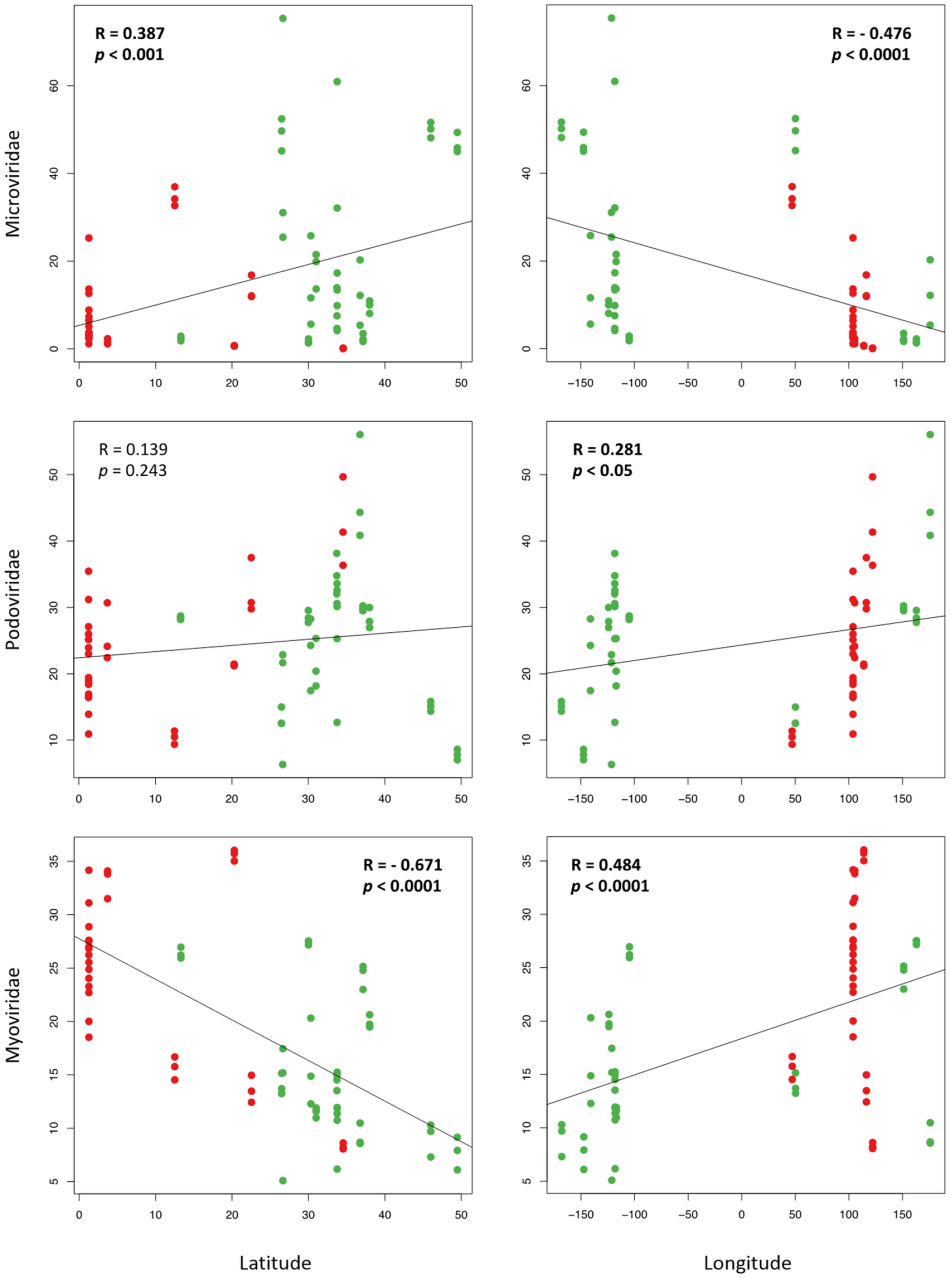
Response of the top three viral families contributing most to the virome dissimilarity between geographical variation. Relationship between relative abundances of *Microviridae*, *Podoviridae*, and *Myoviridae* and samples’ geographic origin was examined. Latitude and longitude are expressed in decimal scale. R was the Pearson correlation coefficient for the relative abundance of viral families against the either latitude or longitude in 72 data sets. Bold text indicates a statistical significance. Green and red dots represent vessels with ballast waters arriving in the Port of LA/LB and the Port of Singapore, respectively.

We next identified that ssDNA phage, *Microviridae* (32.3%) and dsDNA phage, *Podoviridae* (18.1%) and *Myoviridae* (16.0%) contributed most to the virome dissimilarity between geographic origins (S4 Table). Correlation analyses between these phage groups and geographical variation revealed that *Myoviridae* had the strongest relationship with geographic location followed by *Microviridae* (Fig 2). Relative abundance of *Myoviridae* had a highly significant negative correlation with latitude (R = - 0.671, *p* < 0.0001) and a positive correlation with longitude (R = 0.484, *p* < 0.0001). In contrast to the *Myoviridae*, response of *Microviridae* to geographical variation demonstrated a positive correlation with latitude (R = 0.387, *p* < 0.001) and a negative correlation with longitude (R = - 0.476, *p* < 0.0001). Unlike these two phage families, *Podoviridae* had a weak correlation only with longitude (R = 0.281, *p* < 0.05), suggesting that each viral family has different specificity to geographic location. Thus, specific viral families may have unique geographic and environmental niches and these relationships may be masked if better resolution of the genomic diversity is not ascertained.

**Fig 3:**
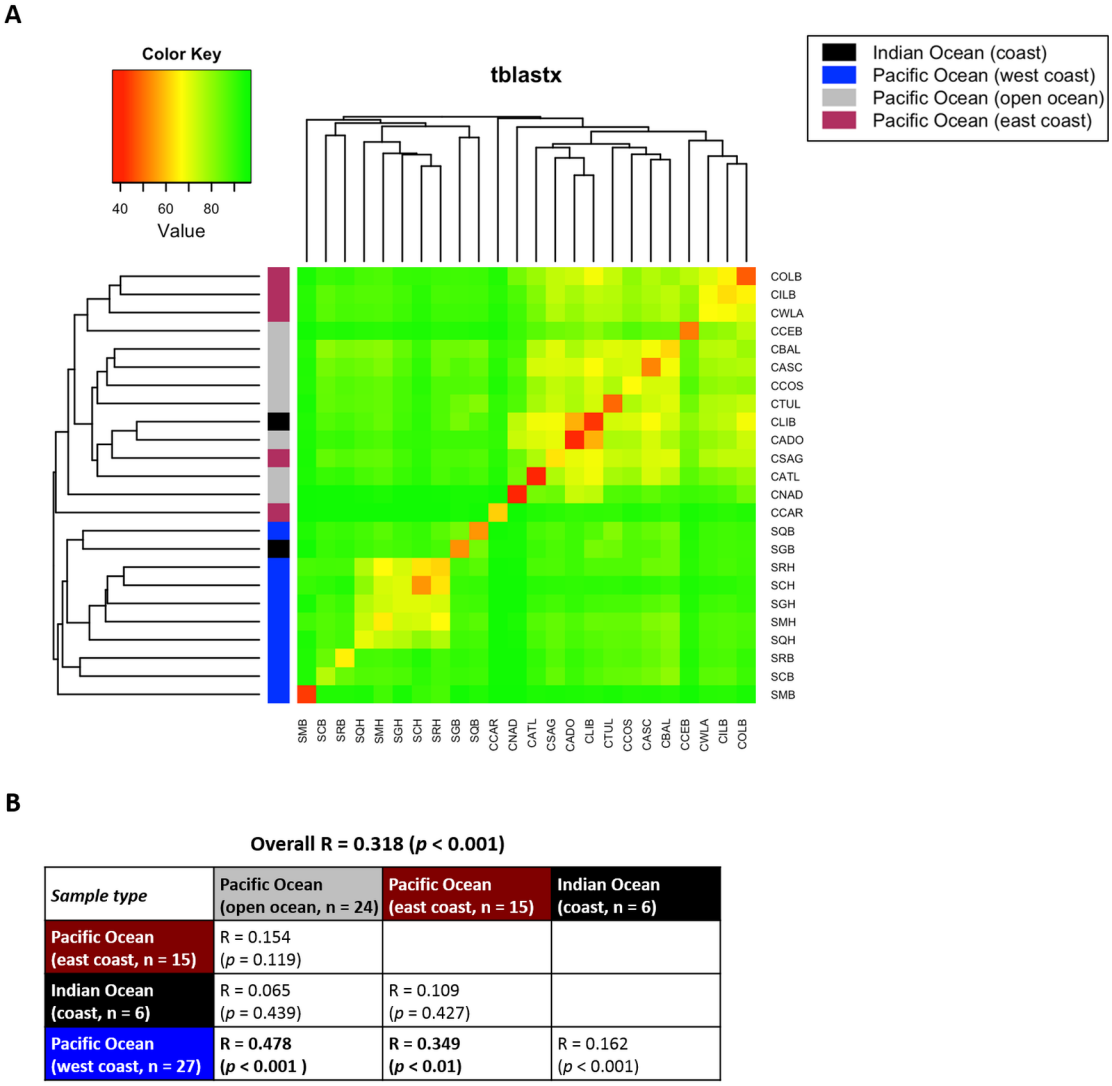
Influence of geography on virome composition. 72 virome data sets were compared with each other based on sequence similarity using TBLASTX comparison. (A) Heatmap presenting the difference in the virome composition. A hierarchical cluster analysis was performed using the complete linkage algorithm. (B) Analysis of similarity result to identify the difference in the virome composition. Bold text indicates a significant difference between ocean viromes.

By examining variation in virome profiles of ballast and harbor waters between geographic locations, we tested our hypothesis that the movement of ballast water across the global shipping network transports the ocean virome. To explain variation in virome composition between geographic locations, all 72 samples were visualized with a heatmap based on a dissimilarity matrix for all virome pairs. Fig 3A showed that viromes from west coast of Pacific Ocean were more similar to each other than those from other ocean realms. The significance of this difference was demonstrated by ANOSIM (R = 0.318, *p* < 0.001) and low ANOSIM R-value was associated with indistinct separation of ballast water samples originating from open Pacific Ocean from the other clusters (Fig 3B). This further suggested that marine viromes are not structured only by geographic patterns but also by local environmental conditions as reported by a recent study [10]. Pairwise comparisons showed that viromes of western Pacific Ocean bordering Eastern Asia were separated from those of either open Pacific Ocean (R = 0.478, *p* < 0.001) or eastern Pacific Ocean (R = 0.349, *p* < 0.01) along the west coast of America, while this seperation was not observed between eastern Pacific Ocean and open Pacific Ocean (R = 0.154, *p* = 0.119).

### Effect of engineered, management, and environmental variables on the ocean virome

**Fig 4:**
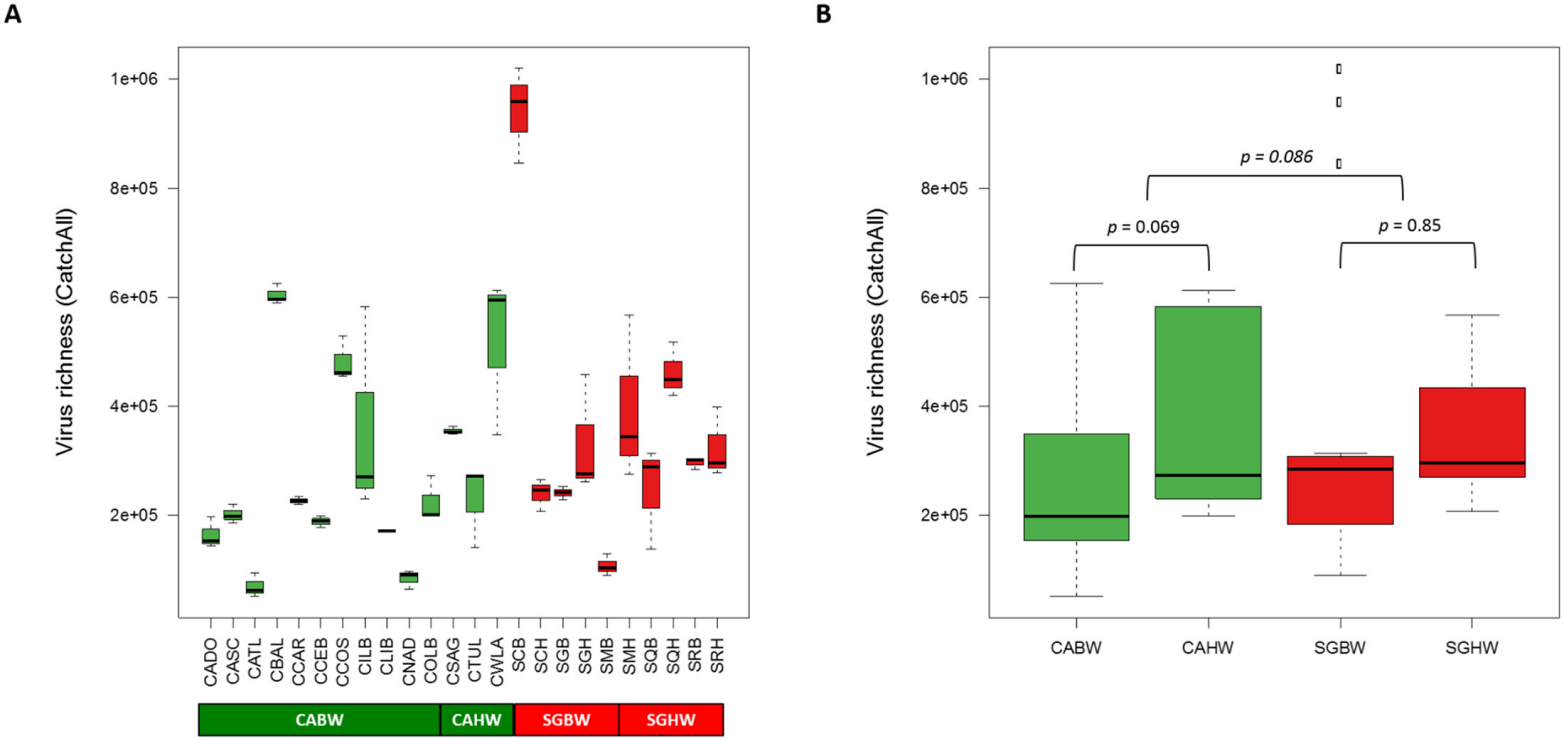
Comparison of virus richness between ballast and harbor waters. (A) Boxplot presenting virus richness of individual sample. (B) Boxplot presenting virus richness of ballast and harbor water groups. Black lines within boxplots represent median values and whiskers indicate minimum and maximum values. CABW, ballast water from the Port of LA/LB; CAHW, harbor water from the Port of LA/LB; SGBW, ballast water from the Port of Singapore; SGHW, harbor water from the Port of Singapore.

Ballast water exchange operation has been considered to be efficient to prevent the introduction of nonindigenous species based on previous findings where lower viral abundances (low number of viral particles) were found in the mid-ocean relative to coastal environments [32–34]. Due to limited ecological protection afforded by ballast water exchange operation, a more stringent ballast water discharge standard has been issued and awaiting additional research and technological advances [35]. This so called ‘Phase 2 standard’ is based on regulating the number of organisms that are discharged with ballast water below the specific limits [36]. Considering the environmental impact of viruses on host population even at a low concentration, however, potential use of viral abundance, which focuses on the number of viral particles, as a regulatory parameter might not meet the goal of preventing viral invasions through ballast water. A better understanding of the types of viruses, that is virus richness in ballast water would improve our ability to assess the risk of exposure of marine fauna and flora to viruses and potentially the risk to humans. We evaluated efficacy of ballast water exchange in reducing the number of different viruses by comparing virus richness (known and unknown viruses calculated by CatchAll) between ballast and harbor waters. Overall, virus richness varied considerably across samples (ranged from 50,745 to 1,020,020) (Fig 4A and S5 Table). The ballast and harbor waters collected from the Port of Singapore (358,362.2 ± 227,906.8) had higher virus richness than those from the Port of LA/LB (276,857.4 ± 169,598.9) (Fig 4B). However, this difference was not statistically significant (*p* > 0.05). When comparing virus richness between ballast and harbor waters at each port, harbor waters had higher virus richness (367,735.8 ± 177,556.4) than ballast waters (252,072.4 ± 161,293.9) in the Port of LA/LB, while ballast waters (366,435.0 ± 308,806.2) had slightly higher virus richness than harbor waters (350,289.4 ± 109,964.7) in the Port of Singapore. These differences were not statistically significant (*p* > 0.05).

**Fig 5:**
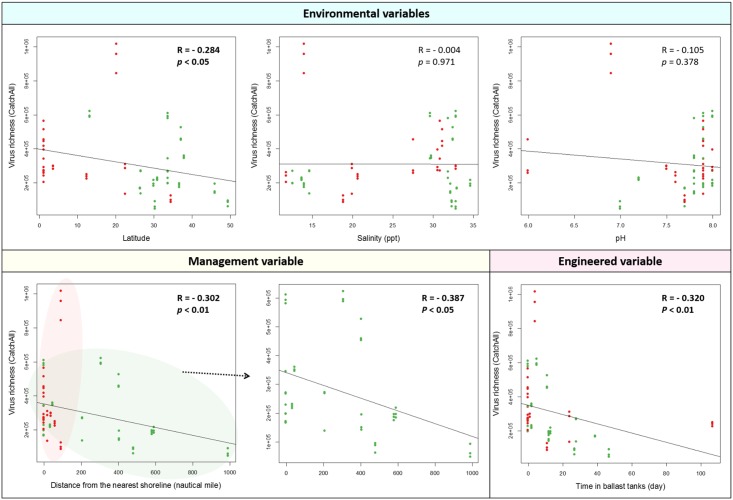
Effect of engineered, management, and environmental variables on virus richness (known and unknown viruses calculated by CatchAll) in ballast and harbor water (*n* = 72). Response of virus richness to engineered, management, and environmental variables was examined. Viral richness estimates for ballast and harbor water viromes were calculated using CatchAll. R was the Pearson correlation coefficient for the virus richness against the variables. Bold text indicates a statistical significance. Green and red dots represent ballast and harbor waters collected from the Port of LA/LB and the Port of Singapore, respectively.

Due to an inconsistent pattern observed between the two ports, we further hypothesized that other variables rather than type of water (either ballast or harbor water) play a more important role in determining virus richness. We first investigated the effect of environmental variables on virus richness (both known and unknown viruses as calculated by CatchAll) in ballast and harbor water. To this end, water temperature, salinity, and pH were selected as they have been reported to be important for virus survival and infectivity [37]. As a vessel approaches a destination port, water temperature in ballast tanks becomes similar to that of surrounding environment. Therefore, latitude of samples’ geographic origin was used as a representative of original water temperature based on the significant relationship between temperature and latitude (R = - 0.743, *p* < 0.0001, S1 Fig). Increased water temperature had a slight negative relationship to virus richness (R = - 0.284, *p* = 0.101), indicating that viruses were present in higher richness near the equator and lower richness at higher latitudes (Fig 5). Either positive or negative correlation did not exist between virus richness and water salinity (R = - 0.004, *p* = 0.971) and pH (R = - 0.105, *p* = 0.378), yet salinity ranges did not include lower levels found in estuaries or freshwater.

As virus richness varied across samples and neither type of water nor environmental variables strongly affected virus richness, we next investigated effect of engineered and management variables on virus richness in ballast and harbor water. As the current ballast water management requires a minimum of 200 nautical miles (1 nautical mile = 1.852 kilometers) from any shoreline to conduct ballast water exchange [38], a significance of distance from shoreline on virus richness was investigated. A correlation analysis using 72 data sets indicated that lower virus richness was shown in ballast water replaced farther from any shoreline (R = - 0.302, *p* < 0.01) (Fig 5). As all vessels arriving in the Port of Singapore did not meet the distance requirement (> 200 nautical miles) of ballast water exchange, significance of distance on virus richness of the samples only from the Port of LA/LB was analyzed to avoid any bias. A statistically significant decrease in virus richness was observed with increased distance from shoreline in samples from the Port of LA/LB (R = - 0.387, *p* < 0.05). This indicated that 200 nautical miles limit was efficient in reducing virus richness of ballast water discharged into the Port of LA/LB. The effect of an important engineered variable, water storage duration in ballast tanks, on virus richness was also investigated. Again, a significant relationship was observed between virus richness and duration of water in ballast tanks (R = - 0.320, *p* < 0.01), suggesting that viruses are susceptible to the environmental conditions in ballast tanks, e.g., lack of light, low oxygen, and temperature fluctuations. In contrast to a previous finding where no significant variation in viral abundance was found over time and before and after ballast water exchange in ballast tanks [39], management or engineered variables was considered to play a major role in determining richness of viruses present in ballast water.

**Fig 6:**
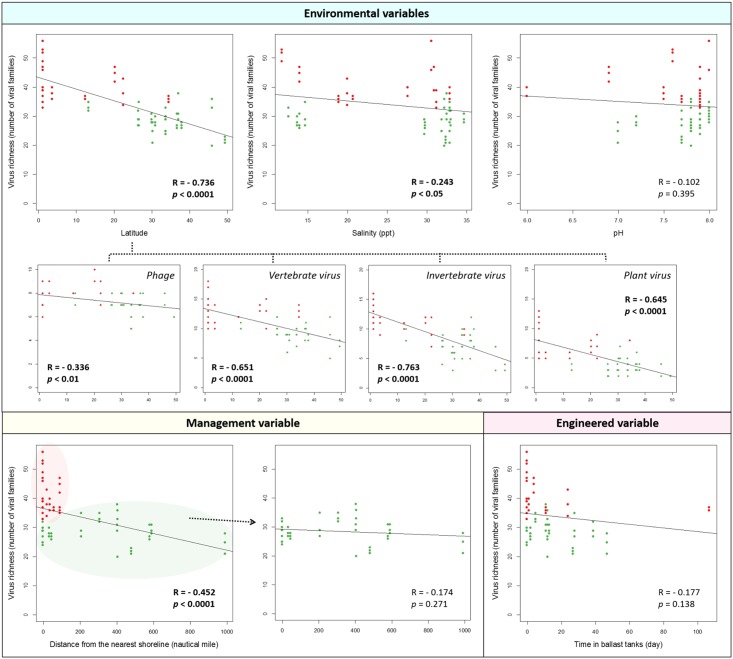
Effect of engineered, management, and environmental variables on virus richness (known viruses defined as a total number of identified viral families) in ballast and harbor water (*n* = 72). Response of virus richness to engineered, management, and environmental variables was examined. Viral richness was defined as a total number of identified viral families in the data sets. R was the Pearson correlation coefficient for the virus richness against the variables. Bold text indicates a statistical significance. Green and red dots represent ballast and harbor waters collected from the Port of LA/LB and the Port of Singapore, respectively.

Assigned taxonomic group is only a small percentage of the metagenomic data sets due to the limitations of the current publically available sequence databases. However, hazard identification is an important question from a public health and environmental disease transmission perspective in virology. Thus, we also investigated effect of engineered, management, and environmental variables on richness of known viruses in ballast and harbor water. A correlation analysis revealed that viruses were present in higher richness near the equator and lower richness at higher latitudes (R = - 0.736, *p* < 0.0001, Fig 6). Furthermore, each host group (e.g., phage, vertebrate virus) showed different degrees of relationship with temperature and the weakest relationship was found in phage group. Importantly, our result suggested restricted geographical distribution of other eukaryotic (including animal and plant) viral groups with strong implications regarding invasion of local biological systems (unlike the homogeneous distribution of phages across the oceans). Increased water salinity had a slight inverse relationship to virus richness but its impact on virus richness was less significant than water temperature (R = - 0.243, *p* < 0.05). Either positive or negative correlation did not exist between virus richness and water pH (R = - 0.102, *p* = 0.395) similar to what is shown in the Fig 5.

While a statistically significant decrease in richness of known and unknown viruses was observed with increased distance from shoreline in samples from the Port of LA/LB (Fig 5), a correlation did not exist between richness of known viruses and distance from shoreline (R = - 0.174, *p* = 0.271, Fig 6). No significant relationship was again observed between richness of known viruses and duration of water in ballast tanks (R = - 0.177, *p* = 0.138), suggesting that management or engineered variables was not playing a major role in determining richness of the rarer known viruses present in ballast and harbor water.

### Potential invasion by rare viral pathogens

**Fig 7:**
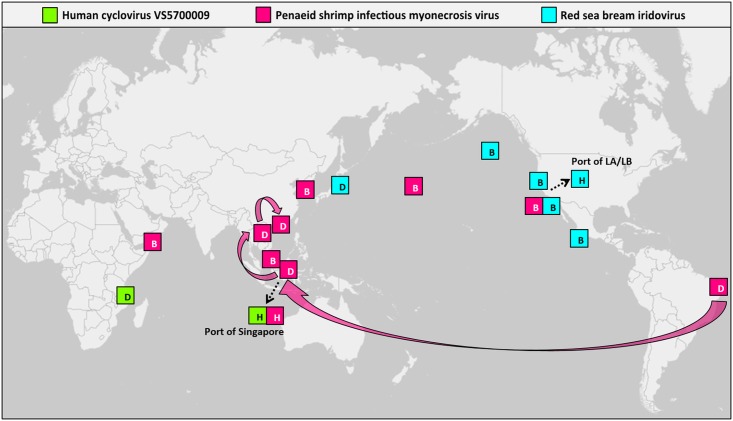
Global distribution of eukaryotic viral pathogens. Samples containing potential viral pathogen-associated contigs were represented in the map. B, ballast water; H, harbor water; D, where viral pathogen-induced disease was found.

Given a significant increase in global ship traffic and its continuous movement of ballast water, we examined the occurrence of potential viral pathogens present in ballast and harbor waters in contrast to where disease in polulations had been identified. In this study, a number of contigs were found to be associated with viruses causing diseases in a wide range of hosts (data not shown). We identified several viral contigs most similar to pathogens infecting human, fish, and shrimp, which were related to significant public health problems or direct economic impact due to reductions in fisheries and aquaculture production (Fig 7 and S6 Table).

In three harbor waters collected from the Port of Singapore, we detected a small ssDNA virus that was closely related to human cyclovirus VS5700009 (CyCV-VS5700009) within the family *Circoviridae*. The translated amino acid seqences of 10 contigs showed best BLASTX matches to replication-associated protein (Rep) (GenBank accession number YP008130363.1) and one contig to capsid protein (Cap) (GenBank accession number YP008130364.1) of viral genome with 88.6% overall amino acid (aa) similarity (ranged from 47.4% to 100%). Genome coverage plot for the CyCV-VS5700009 also confirmed the best matches of contigs onto the Rep and Cap proteins on the reference genome (S2 Fig). Human CyCV-VS5700009 was recently identified in patients with unexplained paraplegia from Malawi by using a metagenomics approach in an attempt to identify unknown human viruses [40]. Together with two subsequent findings of a novel cycloviruses from human samples in Vietnam and Madagascar [41,42], these viruses are considered to be associated with central nervous system infection in humans. Cycloviruses have been found in different sample types from different hosts, including mammals and insects [40] but they have not yet been reported in environmental water samples. Considering strategic location of the Port of Singapore in the heart of Southeast Asia and its connection to numerous ports worldwide, our finding of human CyCV-VS5700009 in the Singapore harbor waters should be noted and the further risk to host populations from this viral pathogen needs to be investigated.

A small icosahedral dsRNA virus that is most closely related to penaied shrimp infectious myonecrosis virus (PsIMNV) was found in the Singapore harbor waters as well as five ballast waters (one from western Asia, two from southeastern Asia, and two from the open Pacific Ocean). PsIMNV is a member of the genus *Giardiavirus* in the family *Totiviridae*. 27 contigs showed best matches to RNA-dependent RNA polymerase (RdRp) (GenBank accession number YP529549.2) with 53.1% overall aa identity (ranged from 22.6% to 85.2%) and three contigs to structural protein (GenBank accession number ABN05324.1) of PsIMNV genome with 64.2% overall aa similarity (ranged from 50.0% to 80.4%). Genome coverage plot for the PsIMNV also confirmed the best matches of contigs onto the RdRp and structural proteins on the reference genome (S3 Fig). PsIMNV has created long-distance distribution in global aquaculture, beginning from Brazil and subsequently spreading to Indonesia, Thailand, and Hainan Province in China [43]. Our finding of PsIMNV in ballast and harbor waters from southeastern Asia was not surprising given the previously reported geographic distribution of PsIMNV. However, the presence of PsIMNV especially in two ballast waters originating from open Pacific Ocean and being discharged in the Port of LA/LB is worthy of close attention as PsIMNV has not been reported in North America.

In four ballast waters whose geographic origins were close to North America as well as harbor waters of the Port of LA/LB, we detected a large dsDNA virus, red sea bream iridovirus (RSIV) belonging to the newest genus *Megalocytivirus* within the family *Iridoviridae*. Nine contigs had homologies with cytosine DNA methyltransferase region of RSIV genome (GenBank accession number BAK14240.1) with 49.5% overall aa similarity (ranged from 44.2% to 59.5%). As Metavir 2 computes genome coverage plots using the NCBI viral database and the RSIV was not listed in the database at the time of analysis, genome coverage plot could not be computed for the RSIV. While RSIV was found in samples whose geographic origins were close to North America in this study, outbreaks of RSIV-induced disease have occurred mainly in Asia [44]. Our result could not reveal epidemiology or transmission patterns of these viral pathogens and further investigations such as gene-specific PCR or phylogenetic approach are also required to confirm the presence of these potential viral pathogens. Nevertheless, our findings of these potential viral pathogens in ballast waters suggested that long-distance distribution of these pathogens could be initiated by continuous movement of ballast water.

## Conclusions

Ballast water is one of the most important vectors for transferring and spreading marine aquatic species throughout the world. Although our understanding of marine viruses (mostly phages) has improved vastly due to technological advancement, factors influencing viral diversity and their fate and transport in marine environments are largely unknown. We used metagenomic tools to provide direct evidence that ballast water harbors a high diversity of viruses and transports them across global marine environments. Driven by international regulations, demand for on-board ballast water treatment approaches has emerged. However, the efficacy of current and novel ballast water treatment methods in reducing or eliminating the potential for virus introduction is largely unexplored. Moreover, significant questions remain in addressing ballast water management challenges, such as which viral pathogens or groups should be targeted or are all viruses equal in their capacity to initiate disease and invasion processes?

We still have much to learn about the geographic distribution of viral species and the role of ballast water as a medium for the spread of invasive viruses. The potential global impact of invasive viruses on marine biogeochemical cycles and ecosystem health warrants further research.

## Supporting Information

S1 Fig Relationship between latitude of samples’ geographic origin and original water temperature. (PDF)

S2 Fig Genome coverage plot for human cyclovirus VS5700009. (PDF)

S3 Fig Genome coverage plot for penaied shrimp infectious myonecrosis virus. (PDF)

S1 Table Summary of sampling information and variables. (XLS)

S2 Table Overview of the sequence reads and the assembled contigs of the virome libraries. (XLS)

S3 Table Summary of virome taxonomic classification. (XLS)

S4 Table Summary of Similarity Percentage (SIMPER) analysis. (XLS)

S5 Table Richness estimates for ballast and harbor water viromes using CatchAll. (XLS)

S6 Table Identified viral pathogens by performing BLASTX searches against non-redundant (nr) database. (XLS)
