# Supplementary figures and images for: Transporting Ocean Viromes: Invasion of the Aquatic Biosphere
Source: PLoS One. 2016 Apr 7;11(4):e0152671. doi: 10.1371/journal.pone.0152671 (PMC4824483; doi:10.1371/journal.pone.0152671)

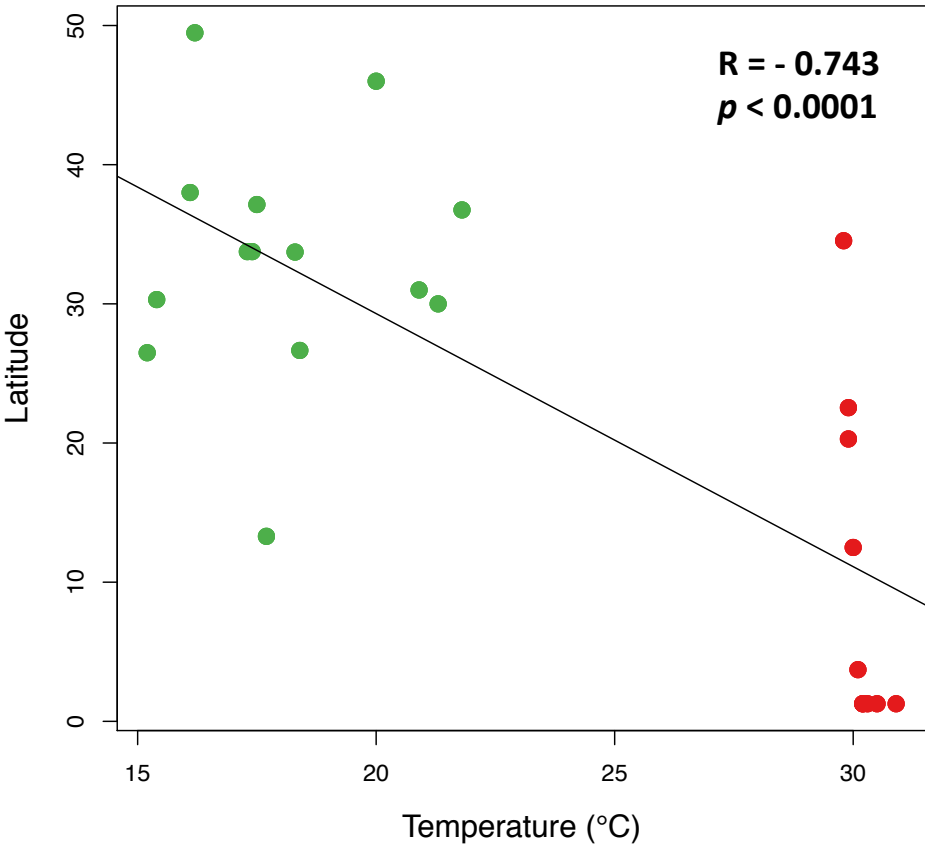

Supplement: S1 Fig — (PDF) [file pone.0152671.s001.pdf]

# Human cyclovirus VS5700009

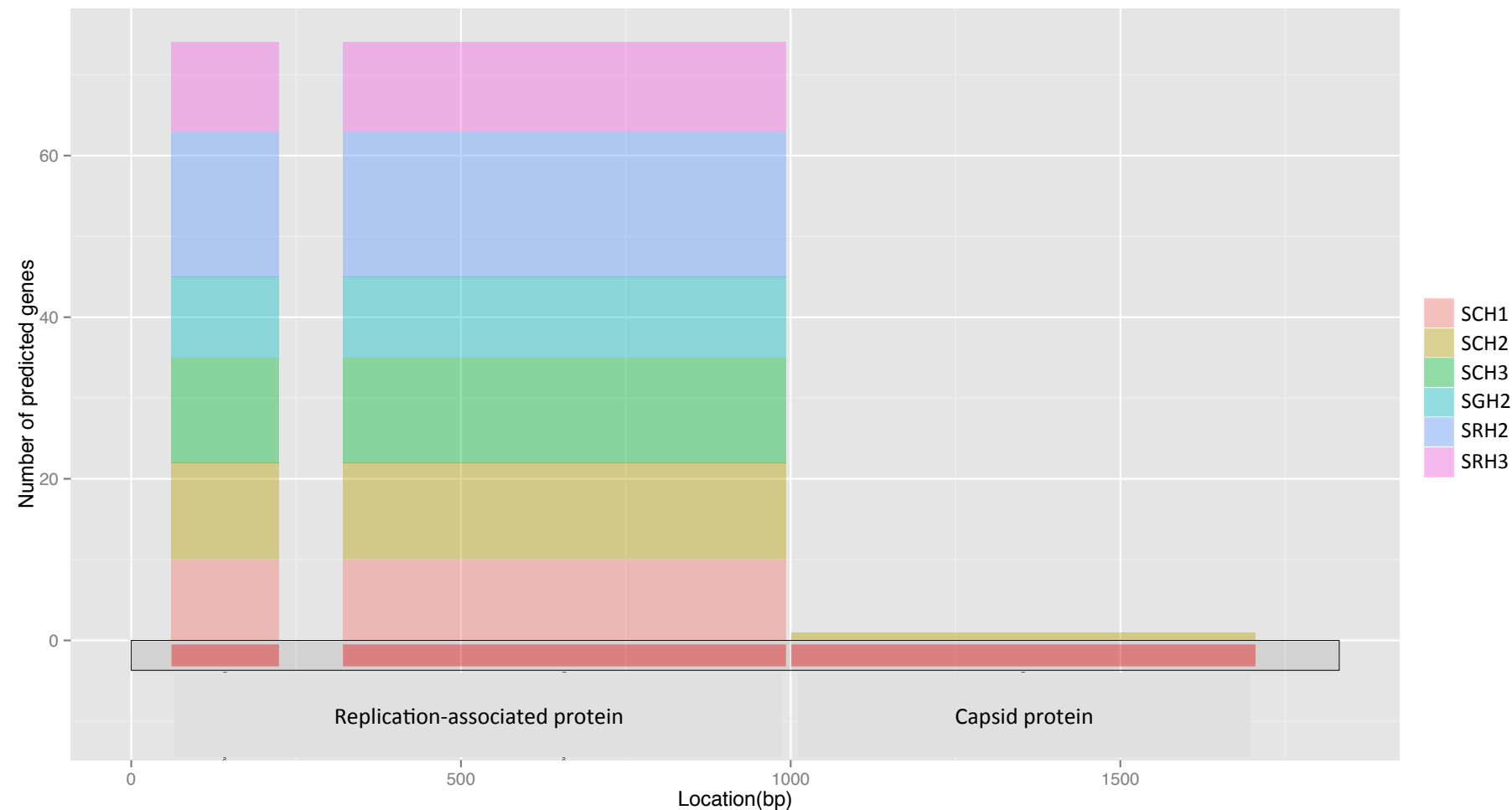

Supplement: S2 Fig — (PDF) [file pone.0152671.s002.pdf]

# Penaied shrimp infectious myonecrosis virus

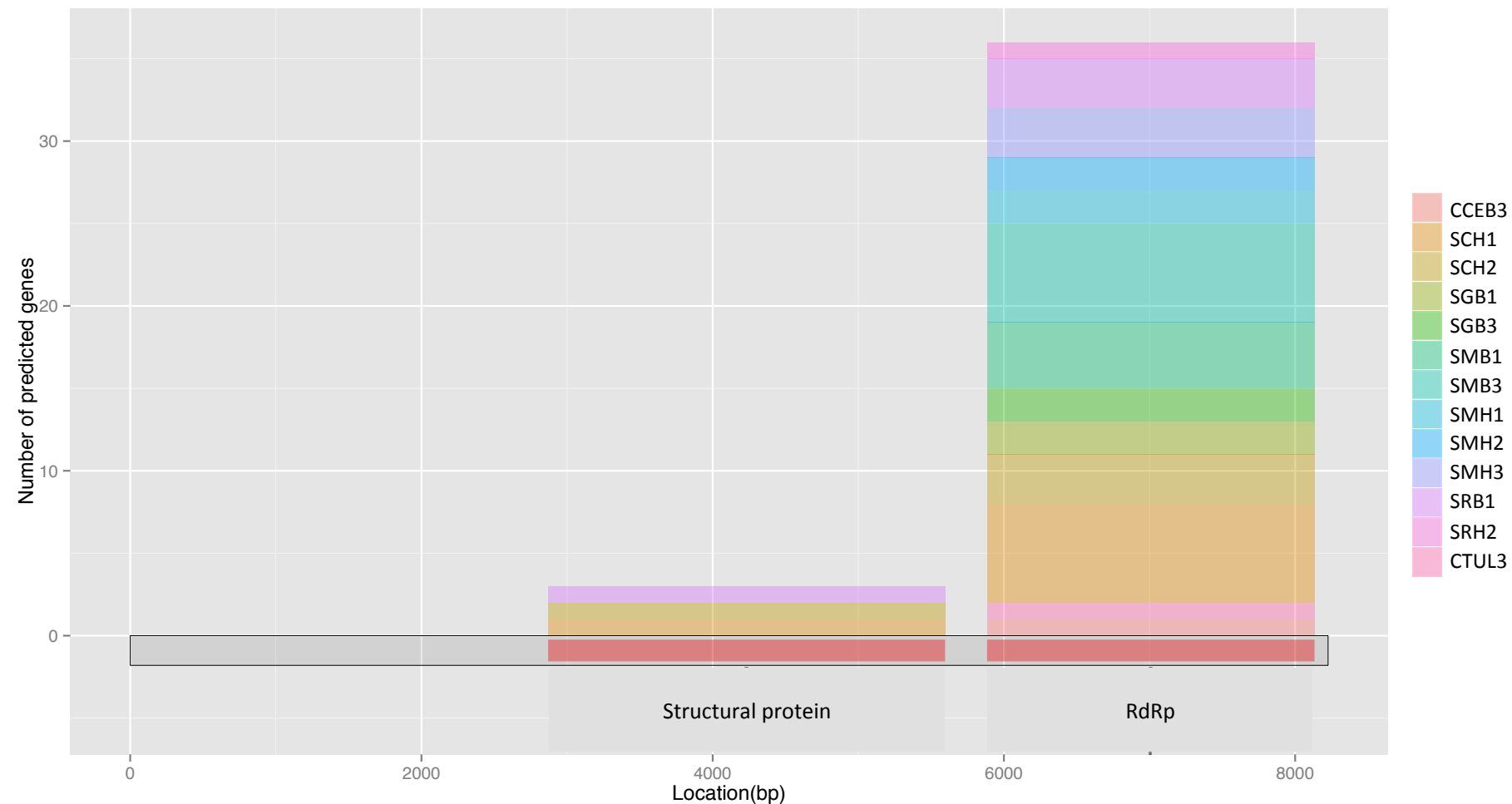

Supplement: S3 Fig — (PDF) [file pone.0152671.s003.pdf]
